# The Positive Association Between Empathy and Self-Esteem in Chinese Medical Students: A Multi-Institutional Study

**DOI:** 10.3389/fpsyg.2019.01921

**Published:** 2019-08-21

**Authors:** Lei Huang, Jessica Thai, Yuan Zhong, Hao Peng, Jessica Koran, Xu-Dong Zhao

**Affiliations:** ^1^Department of Psychiatry, Tongji Hospital, Tongji University School of Medicine, Shanghai, China; ^2^Medical Education Division, Tongji Hospital, Tongji University School of Medicine, Shanghai, China; ^3^College of Medicine, University of Nebraska Medical Center, Omaha, NE, United States; ^4^School of Medicine, Tongji University, Shanghai, China; ^5^Shanghai East Hospital Affiliated to Tongji University, Shanghai, China; ^6^Shanghai Pudong New Area Mental Health Center, Tongji University School of Medicine, Shanghai, China

**Keywords:** empathy, self-esteem, medical student, China, survey

## Abstract

**Background:**

Empathy is an important element of the physician-patient relationship and is a critical personality trait for medical students. However, research has shown that it declines during undergraduate medical education. It is still unclear how empathy interrelates with the psychological elements of medical students, in particular, self-esteem. This study examined the relationship between empathy and self-esteem to explore other possible methods to improve medical students’ empathy.

**Methods:**

A stratified sampling strategy was used to select 1690 medical students from 3 medical institutions in Shanghai as study participants. The questionnaires used to collect data included the Jefferson Scale of Physician Empathy-Student Version (JSPE-S), the Rosenberg Self-esteem Scale (RSES), and a self-made inventory on personal information. Descriptive analysis, independent *t*-test, One-Way ANOVA, and linear regression were used to analyze the data.

**Results:**

The mean empathy score among medical students was 102.73 with SD = 12.64. Multiple regression analysis revealed that, “age,” “perception of the importance of empathy,” “academic pressure,” “desire to be a doctor after graduation,” and “self-esteem” were significant predictors of empathy (*P* < 0.05) and the adjusted *R*^2^ was 0.462. The correlation matrix between empathy and self-esteem was significant (*r* = 0.510, *P* < 0.01). Self-esteem explained 15.5% of the variation of empathy in the final regression model.

**Conclusion:**

There was a positive association between self-esteem and empathy. Self-esteem is one of many factors which contribute to medical students’ empathy. Age, academic pressure, attitude toward empathy and future career also play a critical role in medical student empathy. Enhancing medical students’ self-esteem may be an efficacious way to improve medical students’ empathy.

## Introduction

Over the past 20 years, the patient-physician relationship has been deteriorating in China (Pan et al., 2015; He and Qian, 2016). Many complex reasons contribute to the tense relationship, but the lack of communication skills amongst physicians is an important factor (Blatt et al., 2009). Empathy is an essential component of communication skills and has increasingly become a crucial element for establishing positive patient-health provider relationships (Winefield and Chur-Hansen, 2000; Veloski et al., 2005; Loh and Sivalingam, 2008; Hojat et al., 2013). Studies have evidenced that empathic engagement in patient care leads to improved patient satisfaction and clinical outcomes (Kim et al., 2004; Hojat et al., 2011; Del Canale et al., 2012). Empathetic physicians experience greater job satisfaction, increased health and well-being, and improved clinical decision making (Kim et al., 2004; West et al., 2006; Hojat et al., 2015). Definitions of empathy are diverse. Hojat defines empathy as “a predominantly cognitive attribute that involves an understanding of experiences, concerns, and perspectives of the patient” (Hojat et al., 2002b). Morse considers empathy a construct composed of emotional, moral, cognitive and behavioral dimension (Morse et al., 1992). According to Hemmerdinger et al. (2007), empathy is a personality trait that enables one to identify with another’s situation, thoughts, or condition by placing oneself in their situation. The commonality among these definitions is that empathy is part of a greater psychological domain. Empathy is a crucial element of effective physician-patient communication (Levinson, 1994; Winefield and Chur-Hansen, 2000). It is critical to study the characteristics of students with greater empathy and to encourage empathy development throughout medical training as studies have shown that empathy is considered a “positive personality attribute” (Hojat et al., 2015) and may better predict clinical competence than pre-admission test scores (Stratton et al., 2008).

Socio-demographic and academic factors including gender (Toussaint and Webb, 2005; Schulte-Ruther et al., 2008), year in school (Coulehan and Williams, 2001), and future career preference (Li et al., 2018) affect medical student empathy. Psychological factors such as personality traits (Song and Shi, 2017; Abe et al., 2018), sense of power (Garden, 2009; Toto et al., 2015), stress, and burnout (Rosen et al., 2006; Gleichgerrcht and Decety, 2013; Yuguero Torres et al., 2015) are also influential factors. Several studies worldwide have also shown that women have higher empathy levels than men (Kataoka et al., 2009; Costa et al., 2013; Wen et al., 2013). There has also been evidence suggesting a decline in empathy in medical students in a number of countries as they progress through years of studies, although evidence was mixed (Coulehan and Williams, 2001). Some studies found a reduction (Colliver et al., 2010) in empathy levels during undergraduate education (Austin et al., 2007), while others found no change (Rahimi-Madiseh et al., 2010; Quince et al., 2011; Costa et al., 2013; Toto et al., 2015), or an increase (Kataoka et al., 2009; Magalhaes et al., 2011). Additionally, medical students who preferred not to become doctors had lower empathy than students who preferred to become doctors (Li et al., 2018). Physicians in “people oriented” specialties also consistently had higher empathy scores than those in “technology-oriented” specialties (Romero et al., 2016).

There is limited literature on empathy’s role in medical students’ greater psychological makeup. Although expressing empathy is linked to communication (Winefield and Chur-Hansen, 2000), it also involves a cognitive component with an intent to understand and comprehend others (Berg et al., 2011). In addition to empathy, another psycho-cognitive factor which benefits the patient-healthcare provider relationship is self-esteem (Öhlén and Segesten, 1998), which refers to how favorable an individual’s opinion is of him/herself, positive and negative feelings toward oneself, and his/her personal values (Alkhateeb, 2014). Self-esteem has been strongly correlated with personality traits, affectivity, and extraversion (Watson et al., 2002) as well as self-efficacy (Lane et al., 2004). Individuals with high self-esteem are more capable of handling stress, reducing anxiety and burnout, and developing better communication skills and interpersonal relationships (Kirkpatrick and Ellis, 2006; Edwards et al., 2010). Improved coping mechanisms and increased self-efficacy may mediate a positive relationship between self-esteem and academic performance (Lent et al., 1986; Magnano et al., 2014). While self-esteem and empathy are both influential psychological factors in medical student performance, there have been few studies on the relationship between self-esteem and empathy in medical students. One study by Liu showed a negative relationship between empathy and self-esteem in Chinese medical students (Hanlong, 2012). Other studies on Chinese nursing students and college students had reversed results and suggested that self-esteem was a positive predictor of empathy (Hui, 2002; Hongrui et al., 2016). Another study found that healthcare professionals with higher self-esteem and empathy levels had lower burnout rates (Molero Jurado et al., 2018).

This research study hypothesizes that self-esteem influences medical student empathy. Improving medical students’ self-esteem or discovering mediating factors between self-esteem and empathy may enhance medical students’ empathy. To our knowledge, the correlation between empathy and self-esteem in medical students has not been fully interpreted. Inconsistent past study results may have occurred from study participants spanning across different specialties and from a limited sample size. This research study consequently examines the relationship between self-esteem and empathy in medical students in China, in addition to assessing the differences in empathy scores by gender, year of study, and other academic factors via a multi-institutional design.

## Materials and Methods

### Participants

A total of 1958 medical students were invited to this study and 1690 chose to participate. The response rate was 86.31%. 693(41.01%) students were from Fudan University Shanghai Medical College, 576(34.08%) students were from Shanghai Jiao Tong University School of Medicine, and 421(24.91%) students were from Tongji University School of Medicine. This study used a stratified sampling strategy to select randomly select subjects by inviting 120–150 students to participate from each medical student year from each of the three universities. The sample consisted of 707(41.83%) male and 983 (58.17%) female participants. Participant age ranged from 16–27 years (M = 20.26, SD = 1.622). All participants attended medical school in Shanghai, were unmarried, high school graduates, and most were from single-child families. According to the medical undergraduate education system among the three institutions, medical students were divided into three stages by school year. 785(46.45%) students were in basic medical science courses from first to second year, 603(35.68%) students were in clinical courses from third to fourth year, and 302(17.87%) students were in internships in their fifth year.

### Measures

#### Jefferson Scale of Physician Empathy-Student Version (JSPE-S)

Empathy was measured via the Jefferson Scale of Physician Empathy-Student Version (JSPE-S) (Hojat et al., 2018), which is a validated instrument for use with medical students. The JSPE-S itself is a 20-item questionnaire, 10 items of which are negatively worded and reverse scored and is delivered using a 7-point Likert scale. It is widely used and has been translated in 54 languages, including Chinese (Wen et al., 2013). Students were provided with a statement to which they chose an option between strongly disagree and strongly agree. Possible scores ranged from 20 to 140, with higher scores indicating possibly higher empathy levels (Hojat et al., 2002; Hojat et al., 2003; Sherman and Cramer, 2005). In this study, the Cronbach’s alpha coefficient was 0.838.

#### Rosenberg Self-Esteem Scale (RSES)

This study evaluated self-esteem with the RSES (Rosenberg, 1965), which is a self-rating scale consisting of 10 items, using a 4-point Likert scale to rate, with options ranging from “1 = strongly disagree” to “4 = strongly agree.” The total score ranged from 10 points to 40 points. Higher scores indicated higher self-esteem. The scale was shown to have good reliability and validity in regards to Chinese culture (Zhao et al., 2012; Kong and You, 2013). In this current study, the Cronbach’s alpha coefficient of RSES was 0.857.

#### Self-Reported Personal Information

Personal information was collected, including (1) socio-demographic factors: “gender” with the binary answer “1 = male” and “2 = female,” age; (2) academic factors: “year of school” with the answer “1 = Basic science course (the 1st and 2nd year),” “2 = Clinical course (the 3rd and 4th year),” “3 = Internship (the 5th year)”; “academic pressure” assessed with multiple categorical answer “1 = low,” “2 = moderate,” “3 = Important”; “perception of the importance of empathy” assessed with multiple categorical answer “1 = not important,” “2 = ambivalent,” “3 = important”; “desire to be a doctor after graduation” with the binary answer “0 = no” and “1 = yes”.

### Procedure

Participants were instructed to complete a questionnaire survey including measures of empathy, self-esteem and personal information in the classroom after signing informed consent forms. Three staff from Fudan University Shanghai Medical College, Shanghai Jiao Tong University School of Medicine, and Tongji University School of Medicine administered the survey. It took approximately 15 min for students to complete all the instruments. Each participant received a pen for participating in the study.

### Data Analysis

The Statistical Package for Social Sciences (SPSS; Version 19.0) was used for data storage, tabulation, and the generation of descriptive statistics. Statistical means were used to describe the descriptive data. Independent samples *t*-test and One-Way Analysis of Variance (ANOVA) were used to compare empathy levels among. Groups were divided via self-reported data including gender, year of school, academic pressure, perception of the importance of empathy, and desire to become a doctor after graduation. Pearson correlation and multivariate stepwise regression were conducted to explore how levels of empathy and self-esteem as well as other personal factors were related. All tests were two tailed unless otherwise stated. Results were considered statistically significant if the *p* value was <0.05.

## Results

### Descriptive Analysis of Empathy and Self-Esteem

As shown in Table 1, mean empathy level was M_*empathy*_ = 102.73, SD_*empathy*_ = 12.64. With regard to the three subscales, mean “perspective taking” score was M_*PT*_ = 54.75, SD_*PT*_ = 7.78, mean “compassionate care” score was M_*cc*_ = 39.78, SD_*cc*_ = 6.05, and mean “standing in patient shoes” scores was M_*SISS*_ = 8.20, SD_*SISS*_ = 2.46. Mean self-esteem assessed by RSES score ranged was M_*SE*_ = 31.23, SD_*SE*_ = 4.08.

**TABLE 1:** Descriptive analysis of JSPE-S and RSES (*n* = 1690).

**Variables**	**Mean**	***SD***	**Min**	**Max**
JSPE-S	102.73	12.64	60	140
Perspective taking	54.75	7.78	31	70
Compassionate care	39.78	6.05	12	56
Standing in the patient’s shoes	8.20	2.46	2	14
RSES	31.23	4.08	18	40

### Correlation Between Empathy and Self-Esteem as Well as Personal Variables

Independent samples *t*-test and One-Way ANOVA showed significant correlations among empathy and all of the personal variables defined in this study, including “gender” (*t* = −2.908, *p* = 0.004), “year of school” (*F* = 11.467, *p* < 0.001), “perception of the importance of empathy” (*F* = 168.321, *p* < 0.001), “academic pressure” (*F* = 7.685, *p* < 0.001), and “desire to be a doctor after graduation” (*t* = 4.891, *p* < 0.001) (Table 2). The correlation matrix between empathy and age (*r* = −0.130, *p* < 0.01) as well as self-esteem (*r* = 0.510, *p* < 0.01) were statistically significant (Table 3).

**TABLE 2:** Comparison of the level of empathy among groups with different personal and academic variables.

**Variables**	***n***	**%**	**JSPE-S *Mean***	**JSPE-S *SD***	**t/F**	***p***
Gender						
Male	707	41.83	101.68	13.19	–2.908	0.004
Female	983	58.17	103.49	12.17		
Year of school						
Basic science course (1st and 2nd)	785	46.45	103.92	12.85	11.467	*p* < 0.001
Clinical course (3rd and 4th)	603	35.68	102.64	12.31		
Internship (5th)	302	17.87	99.85	12.29		
Academic pressure						
Low	67	4.00	97.40	13.99	7.685	*p* < 0.001
Moderate	861	50.90	102.46	12.76		
High	762	45.10	103.52	12.25		
Perception of the importance of empathy						
Not important	49	2.90	82.24	8.36	168.321	*p* < 0.001
Ambivalent	163	9.54	91.91	9.19		
Important	1478	87.46	104.61	11.86		
Desire to be a doctor after graduation						
Yes	1588	94.00	103.11	12.54	4.891	*p* < 0.001
No	102	6.00	96.84	12.76		

**TABLE 3:** Correlation analysis between empathy and self-esteem.

**Variables**	**Perspective taking**	**Compassionate care**	**Standing in the patient’s shoes**	**JSPE-S**
Age	−0.116^∗∗^	−0.093^∗∗^	−0.068^∗∗^	−0.130^∗∗^
RSES	0.430^∗∗^	0.418^∗∗^	0.230^∗∗^	0.510^∗∗^

*^∗∗^*p* < 0.01.*

### Multivariate Regression Analysis

To investigate the relationship between students’ empathy and a single factor with marked discrepancy, we took the sum of JSPE-S scores as the dependent variable, and “gender,” “age,” “year of school,” “academic pressure,” “perception of the importance of empathy,” “desire to be a doctor after graduation,” and “self-esteem” as the independent variables. A linear regression and the stepwise method were used to determine the main factors (Stepwise Criteria: Probability-of-F-to-enter ≤0.050, Probability-of-F-to-remove ≥0.100). Of the three models, the third had the highest *R*^2^ value (0.468) and was consequently selected as the final model. Furthermore, we found that “age,” “perception of the importance of empathy,” “academic pressure,” “desire to be a doctor after graduation,” and “self-esteem” were significantly predictors of empathy (*p* < 0.05). Moreover, “age” showed justified negative correlations with empathy, while the others showed positive correlations (Tables 4, 5).

**TABLE 4:** Stepwise model summary between empathy and the single factor with marked discrepancy.

**Model**	***R***	***R*^2^**	**Adjusted *R*^2^**	**Standard error of the estimate**	**Change statistics**				
					***R* change**	***F* change**	**df1**	**df2**	***p* change**
(1)	0.148^*a*^	0.022	0.021	12.504	0.022	18.976	2	1687	0.000
(2)	0.556^*b*^	0.309	0.307	10.523	0.287	174.732	4	1683	0.000
(3)	0.681^*c*^	0.464	0.462	9.271	0.155	486.182	1	1682	0.000

*^*a*^ Predictors: (Constant), gender, age. ^*b*^ Predictors: (Constant), gender, age, year of school, academic pressure, perception of the importance of empathy, desire to be a doctor after graduation. ^*c*^ Predictors: (Constant), gender, age, year of school, academic pressure, perception of the importance of empathy, desire to be a doctor after graduation, self-esteem.*

**TABLE 5:** Hierarchical linear regression of empathy and the single factor with marked discrepancy.

**Explanatory Variables**		**Model 1**			**Model 2**			**Model 3**		
		
		**β (95%CI)**	***F***	***p***	**β (95%CI)**	***F***	***p***	**β (95%CI)**	***F***	***p***
Socio-demographic factor	Gender Age	0.072 (0.643, 3.062) −0.130(−1.384, −0.648)	3.003 −5.418	0.003 0.000	0.044(0.114, 2.157) −0.046(−0.896, 0.173)	2.179 −1.326	0.029 0.185	0.031 (−0.104, 1.697) −0.081 (−1.105, −0.162)	1.735 −2.633	0.083 0.009
Academic factors	Year of school				0.010(−0.989,1.322)	0.282	0.778	0.014(−0.0775, 1.261)	0.469	0.639
	Academic pressure				0.024 (−0.360, 1.426)	1.170	0.242	0.072 (0.805, 2.390)	3.953	0.000
	Perception of the importance of empathy				0.530 (8.021, 9.354)	25.567	0.001	0.424(6.351, 7.565)	22.485	0.000
	Desire to be a doctor after graduation				0.087 (2.510, 6.734)	4.292	0.001	0.054 (0.994, 4.729)	3.006	0.003
Self-esteem								0.411 (1.158, 1.385)	22.050	0.000
Corrected Model			18.976	0.000^b^		125.419	0.000^c^		207.948	0.000^d^
Adjusted *R*^2^			0.021			0.307			0.462	

*^*b*^Predictors: (Constant), gender, age. ^*c*^Predictors: (Constant), gender, age, year of school, academic pressure, perception of the importance of empathy, desire to be a doctor after graduation. ^*d*^Predictors: (Constant), gender, age, year of school, academic pressure, perception of the importance of empathy, desire to be a doctor after graduation, self-esteem.*

## Discussion

Empathy has been considered the royal road to an optimal physician-patient relationship and overall physician competence in China (Blatt et al., 2009). In this study, the mean score for medical students in Shanghai (M = 102.73) was similar to previous studies on medical students of one institution in Shanghai (M = 104.2) (Li et al., 2018). However, scores were slightly lower than a study by Wen from Liaoning province (M = 109.60) (Wen et al., 2013). This may be explained by the study participants’ differences in academic year. In Wen’s study, participants were in their first to fourth years in medical school, and the study excluded internship medical students, which may have influenced mean empathy scores. Nonetheless, the overall empathy level of Chinese medical students was significantly lower than that of American medical students (Hojat et al., 2002a). This may be due to cultural differences. It has been suggested that factors such as decreased physician to patient ratios or an overabundance of patients may result in increased burnout and decrease empathy (Diez-Goni and Rodriguez-Diez, 2017). With China’s immense patient population, Chinese medical students may feel overwhelmed and forced to rush through clinical diagnoses and patient visits, leading to distress and lower empathy levels. Furthermore, the increasing use of technology in medicine in China may dehumanize health care providers from empathizing with patients (Li et al., 2018). At the same time, with the wide range of Chinese socioeconomic classes (Bian et al., 2005), Chinese medical students may feel superior to some of their patients, leading to mechanized, emotionally detached medicine (Li et al., 2018). This is concerning because the developmental course of empathy in Chinese medical students across their careers is poorly understood. There may be a possible decrement in empathy across Chinese medical education. A more sophisticated understanding of empathy in medical students is needed, with attention to issues that may adversely impact this crucial aspect of their development (Mahoney et al., 2016).

“Self-esteem” was the second most important predictor of empathy in our study, which was consistent with a study on Chinese nursing students (Hongrui et al., 2016) and college students (Hui, 2002) which revealed that participants with higher RSES scores received higher JSPE-S scores. However, this study contradicted Liu’s study on Chinese medical students (Hanlong, 2012), which showed that self-esteem was negatively correlated with medical students’ empathy. In Liu’s study, individuals with higher self-esteem tended to identify with individuals with their own values and could not accept others’ opinions and feelings. Research has indicated that empathy has been linked, theoretically and empirically, to several psychological attributes, such as personality, stress, anxiety, and burnout. Similarly, self-esteem may affect empathy by these same attributes as self-esteem is correlated with personality and one’s value system (Iacobucci et al., 2013). Previous studies have shown that self-esteem was strongly negatively correlated with neuroticism/negative affectivity and moderately to strongly related to extraversion/positive affectivity (Watson et al., 2002). Individuals with high self-esteem may be more positive and optimistic and have a good sense of security, self-control, and motivation. These feelings and personality traits may consequently help stress management and reduce anxiety or dissatisfaction with life (Xiangkui and Lumei, 2005; Fangsong, 2006). Healthier mentality and positive emotion may also improve the development of better interpersonal relationships (Kirkpatrick and Ellis, 2006), while low self-esteem may lead to emotional problems such as anxiety and sociophobia, which negatively influence interpersonal relationships (Edwards et al., 2010).

Furthermore, stress and anxiety have been shown to lead to occupational burnout (Cass et al., 2016; Youssef, 2016; Zhou et al., 2016; Patel et al., 2017), which may significantly reduce medical students’ empathy (Thomas et al., 2007; Gleichgerrcht and Decety, 2013; Yuguero Torres et al., 2015). According to Alvaro’s conservation of resources theory, accumulating condition resources such as increased well-being and lower rates of burnout may allow greater access to other personal resources. Self-esteem was defined as one of these personal resources which alleviated burnout (Alvaro et al., 2010). Additionally, initial levels of empathy for patients may lead to increased patient satisfaction and positive feedback from patients. This may create a self-perpetuating positive reinforcement cycle, which continues to increase health care providers’ empathy levels (Pollak et al., 2011). In addition to higher patient satisfaction, an additional explanation for positive reinforcement of patient empathy levels is the association between empathy, higher communication, and interpersonal skills, which allow physicians to easily express empathy, create better physician-patient relationships, and receive positive reinforcement to continue expressing empathy (Winefield and Chur-Hansen, 2000; Pollak et al., 2011). It has been suggested that self-esteem is linked with skilled communication (Carson et al., 2001). Individuals with better communication skills and higher self-esteem may be more adept at expressing empathy, leading to greater patient satisfaction and subsequently, an increased desire to continue expressing empathy in the future. This suggests that medical students with greater communication and interpersonal relationship training may be more comfortable expressing empathy (Winefield and Chur-Hansen, 2000; Pollak et al., 2011).

In the final model of regression, “perception of the importance of empathy” was the most significant predictor of empathy. “Academic pressure” as well as “desire to be a doctor after graduation” were also positive but weak contributors of medical students’ empathy. These results suggest that medical students who had a positive attitude toward empathy and future career had higher empathy scores, which echoes a study by Li et al. (2018) which found that medical students who did not want to become physicians had lower empathy scores than medical students who were still unsure of their passion to become physicians. This may be explained by differences in Chinese vs. American medical education. Chinese medical students choose their specialty after senior middle school graduation. Some students do not choose medicine as their career voluntarily, and they may have little interest in medicine and low motivation to become a doctor after their studies (Wang et al., 2014). In contrast, American students must finish their relative undergraduate degree (biology, etc.) before entering medical school and may pursue medicine as a future career with more mature, intentional thought. Additionally, only students with distinct, desirable personality traits such as high empathy may garner acceptance into such competitive American medical schools (Lumsden et al., 2005). Furthermore, medical school curriculum is quite different between China and the United States. While empathy has been recognized as an important ability for medical students, it is still not integrated into most Chinese medical school curriculum (Wen et al., 2013). On the other hand, empathy is objectively assessed on the United States USMLE, which may increase medical students’ emphasis on empathy (Hojat et al., 2002a) and may pressure students to be more empathetic.

Bivariate analysis showed that empathy scores of participants decreased with increasing years of medical training and were lowest during the final clerkship year. Female medical students had greater empathy scores than male participants. However, after controlling for gender and age in the regression analysis, gender and “year of school” did not significantly affect empathy. This hinted that the significant effects seen in the bivariate analysis may be because of other factors which were not controlled for in the multivariate analyses. Although the associations between gender and “year of school” were insignificant after adjusting for some covariates, previous studies illustrated that factors such as gender and “year of school” may affect empathy (Coulehan and Williams, 2001). Therefore, future investigations should include surveys with larger sample sizes and more detailed participant information.

Age was a weak negative predictor of empathy in this study which may be because younger age is associated with increased empathy from less burnout and fatigue in the medical profession (DiLalla et al., 2004). Another explanation may be that older age is correlated with years of clinical training in medical school. United States medical students often take gap years between undergraduate education and medical school and maybe of various ages at medical school admission. On the other hand, it is common in China to matriculate into medical school immediately after high school. As such, Chinese first year medical students are typically younger than second and third year medical students (Chen et al., 2010). As older students gain clinical experience in medical school, they may emotionally harden to being exposed to pain, death, and hardships to mentally protect themselves (Coulehan and Williams, 2001). Likewise, even in the United States, where medical student age may not correlate as well with years of clinical training as in China, older individuals may have accumulated more life experience regarding suffering and may become jaded more easily than younger individuals (Eysenck et al., 1985).

Empathy education is a vital part of medical students’ empathy development. Chinese medical students’ low empathy in this study suggests that current education patterns need improvement. Furthermore, our research found a positive relationship between self-esteem and empathy, as well as academic attitude and empathy. These results suggest that factors decreasing self-esteem or mediating factors such as personality, stress or burnout may subsequently negatively affect empathy levels. Improving medical students’ professional identity as well as increasing educational emphasis on empathy may help increase medical students’ self-esteem and empathy.

### Strengths and Limitations

To our knowledge, this is the first multi-institutional study on Chinese medical students’ self-esteem and empathy. However, we also acknowledge that participants were medical students solely from Shanghai, who were unmarried, mainly from single-child families, and ranged from ages 16–27. This sample is not necessarily representative of empathy levels among medical undergraduates nationwide. Furthermore, participants responded via self-report questionnaires. While these instruments have been validated for the Chinese population, the use of self-reported assessments and the retrospective study design may decrease data validity. Additionally, as this study is cross-sectional and descriptive, future research should confirm our findings via a longitudinal and prospective study. Finally, although the relationship between self-esteem and empathy was positively correlated in this study, there is still a need for further investigation of possible mediating factors such as stress, anxiety, burnout, or wellness.

## Conclusion

Empathy is a particularly important disposition for medical students and toward their future careers as doctors. Self-esteem is one of many influential factors toward medical students’ empathy. This study revealed that there is a positive association between self-esteem and empathy. School year, academic pressure, attitude toward empathy, and desire to be a doctor after graduation are also positively correlated with empathy level while age is indirectly related to empathy. By increasing self-esteem and empathy in medical students and physicians, the quality of the patient-physician relationship may be improved, resulting in reciprocally beneficial outcomes. Patients may become more trusting of physicians and be more open to disclosing their concerns to empathetic physicians. Likewise, empathetic, high self-esteem physicians may be more adept at handling sensitive, emotional patient situations. As such, improving self-esteem and empathy in medical students and physicians is a critical component of providing quality health care. Further research may explore additional mediating factors between self-esteem and empathy. Future studies may also investigate the relationship between self-esteem and empathy in medical students outside of Shanghai such as in more rural cities or in different regions of China. This study may also be replicated outside of China to investigate if there is a cultural influence on self-esteem and empathy.

## Data Availability

All datasets generated for this study are included in the manuscript and/ro the Supplementary Files.

## Ethics Statement

Our study had an ethics approval from the School of Medicine Ethics Committee, Tongji University School of Medicine, Shanghai, China and we acquired written informed consent from study participants.

## Author Contributions

LH contributed to study design, recruitment of participants, data analysis and interpretation and writing of the manuscript. JT and JK assisted in the interpretation of the results and draft writing. YZ and HP assisted in the data acquisition and recruitment of participants. X-DZ contributed to study design and supervision. All authors have approved the final manuscript.

## Conflict of Interest Statement

The authors declare that the research was conducted in the absence of any commercial or financial relationships that could be construed as a potential conflict of interest.

## Supplementary Material

DATA SHEET S1 The Positive Association between Empathy and Self-esteem in Chinese Medical Students.
